# Sexually selected lip colour indicates male group-holding status in the mating season in a multi-level primate society

**DOI:** 10.1098/rsos.150490

**Published:** 2015-12-16

**Authors:** Cyril C. Grueter, Pingfen Zhu, William L. Allen, James P. Higham, Baoping Ren, Ming Li

**Affiliations:** 1 School of Anatomy, Physiology and Human Biology, The University of Western Australia, Crawley/Perth, Western Australia 6009, Australia; 2 Key Laboratory of Animal Ecology and Conservation Biology, Institute of Zoology, Chinese Academy of Sciences, Beijing 100101, People's Republic of China; 3 School of Biological, Biomedical and Environmental Sciences, University of Hull, Hull HU6 7RX, UK; 4 Center for the Study of Human Origins, Department of Anthropology, New York University, New York, NY 10003, USA

**Keywords:** sexual selection, coloration, reproductive seasonality, multilevel society, primate, *Rhinopithecus*

## Abstract

Sexual selection typically produces ornaments in response to mate choice, and armaments in response to male–male competition. Unusually among mammals, many primates exhibit colour signals that may be related to one or both processes. Here, we document for the first time correlates of facial coloration in one of the more brightly coloured primates, the black-and-white snub-nosed monkey (*Rhinopithecus bieti*). Snub-nosed monkeys have a one-male unit (OMU) based social organization, but these units aggregate semi-permanently into larger bands. This form of mating system causes many males to become associated with bachelor groups. We quantified redness of the prominent lower lip in 15 males (eight bachelors, seven OMU holders) in a group at Xiangguqing, China. Using mixed models, our results show that lip redness increases with age. More interestingly, there is a significant effect of the interaction of group-holding status and mating season on redness; that is, lip colour of OMU males undergoes reddening in the mating season, whereas the lips of subadult and juvenile bachelor males become paler at that time of year. These results indicate that lip coloration is a badge of (group-holding) status during the mating season, with non-adults undergoing facial differentiation, perhaps to avoid the costs of reproductive competition. Future research should investigate whether lip coloration is a product of male–male competition, and/or female mate choice.

## 1. Introduction

Darwin identified two major processes of sexual selection—intrasexual selection, which favours traits that enable individuals of one sex to outcompete rivals for mating opportunities with the opposite sex, and intersexual selection, which favours traits that make individuals of one sex more attractive to the other [1]. In mammals, intrasexual selection has been strong on males and has led to highly armoured males exhibiting weaponry that facilitates male–male competition, such as horns, antlers and large canines. By contrast, intersexual selection has been strong in other taxa, such as birds, leading to highly ornamented males who exhibit colourful crests, extravagant tail feathers and brightly coloured beaks, aimed at attracting females [2–5].

Unusually for mammals, some primates are noteworthy for displaying brightly coloured skin. At first glance, these colourful ornaments might seem analogous to those of birds and fish, and one might therefore assume that they are primarily aimed at attracting females. However, a number of studies of primate species have shown that they indicate mate social status and are used in male–male agonistic interactions, suggesting that like many mammalian sexually selected traits, they have been selected primarily through male–male competition (e.g. mandrills [6,7], geladas [8], drills [9], vervet monkeys [10]). For males, such signals may provide information on the social status of the rival, and the potential risks associated with engaging in a contest with a particular competitor, allowing imminent conflicts to be resolved without the need for escalation. Whether these signals are also important in female mate choice is less clear. In rhesus macaques, male coloration does not correlate with social status [11,12] and instead appears to be used in female mate choice [11]. In mandrills, there is evidence that male coloration may be involved in both male–male competition and female mate choice processes [13]. However, a study of drills only found evidence that male coloration is involved in indicating social status, not in attracting females beyond the effects of dominance rank [9].

**Figure 1.**
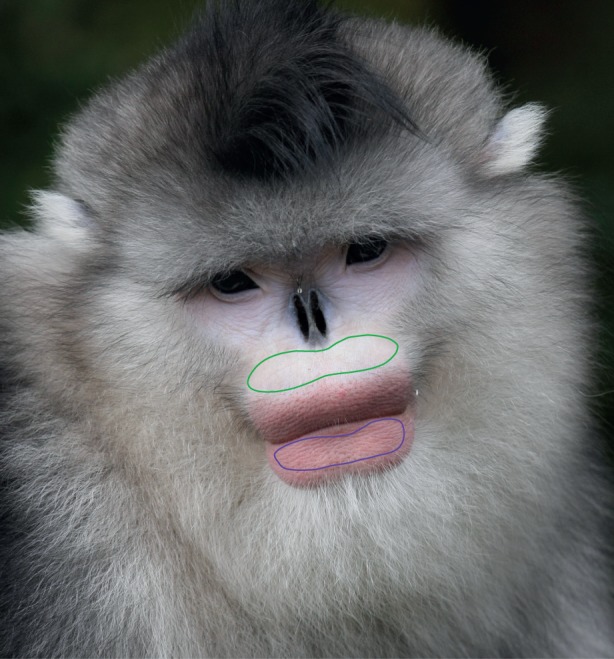
Example of the face and lip regions selected for analysis. The lip colour was divided by the face colour, effectively standardizing different images against face colour thus controlling for different lighting and photographic set-ups between images.

**Figure 2.**
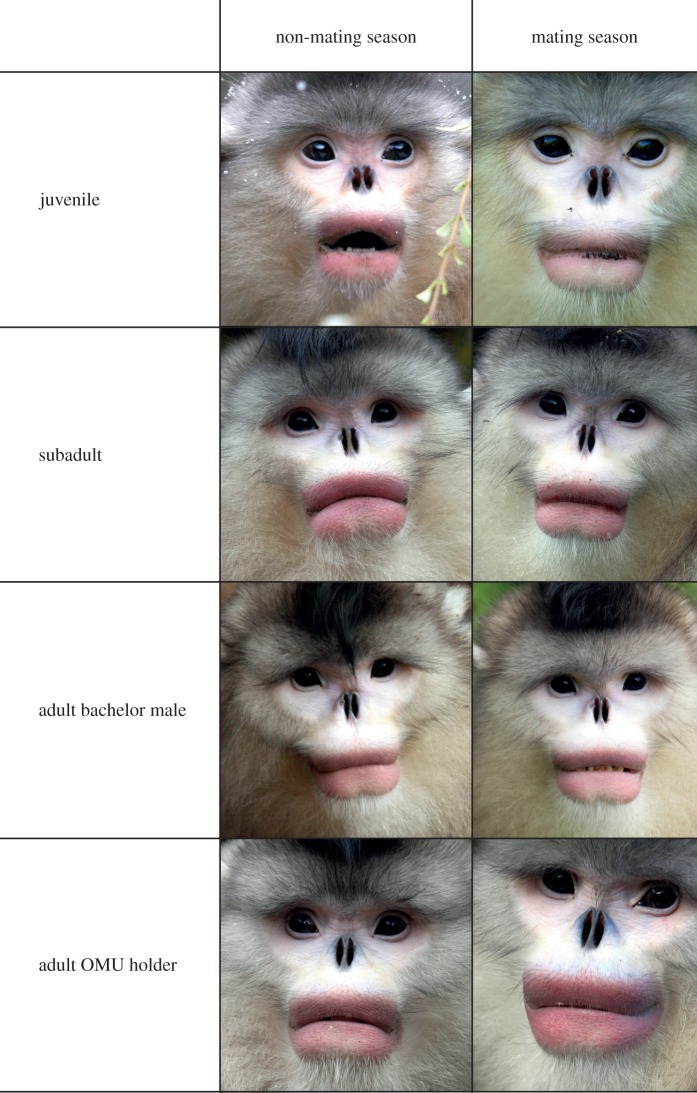
Examples of the range of lip colour variation in different age-sex classes. Each row shows one individual in the non-mating and mating season.

Snub-nosed monkeys (genus *Rhinopithecus*) are a primate group that displays notable levels of ornamentation and facial coloration (figures 1 and 2) but in which the function of this coloration is unstudied. Based on our understanding of coloration in other primate groups, they therefore enable a test of predictions and our state of knowledge on the function of male primate colour signals. They are also an interesting group in which to address the evolution of such signals for several reasons. First, they exhibit a one-male unit (OMU)-based social organization, but in contrast to most other colobines these units aggregate semi-permanently into larger bands (modular or multilevel societies) [14]. This creates a situation in which male–male sexual competition is more frequent than in species without modularity, as evidenced by greater levels of sexual dimorphism in body mass [15]. Comparative research has also shown that primates in multilevel systems score higher on a Likert scale of dimorphism in ornamentation than species in other types of social system [16].

Second, these bands can reach extreme sizes of up to 500 individuals [17], thus necessitating means of quality assessment that do not rely on individual recognition. Colour ornaments are thought to be particularly important in large social groups in which individual recognition may be limited, and in which they might facilitate the assessment of an individual's characteristics to conspecifics [8,18]. Indeed, studies have shown that group size is a strong predictor of complexity of facial colour patterns in catarrhine primates [19] and levels of ornamental dimorphism in anthropoid primates [16].

Third, while copulations have been reported year-round, there is a peak in mating activity during a seasonally restricted period (i.e. a mating season) [20,21]. In some catarrhines, including rhesus macaques [22] and crested macaques [23], male coloration undergoes seasonal fluctuation with increases in expression when a greater number of females are fertile. This could reflect an increase in competition with other males at this time (and so still be related to a function of coloration in male–male competition), or it could indicate a role for coloration in attracting females.

Fourth, males can be categorized as being associated with bachelor groups (all-male units, AMUs) or OMUs. Young males are expelled from their natal units and subsequently join bachelor groups [24] which are almost constantly in relatively close proximity to the band of OMUs [25]. Bachelor males usually do not have reproductive access to band females. A study on geladas, who exhibit very similar modular societies, has shown that OMU leaders have redder chests than bachelor males [8].

Here, we test our current understanding of the function of male coloration in primates by assessing the correlates of male coloration in black and white snub-nosed monkeys (*Rhinopithecus bieti*). We collected data on male lip coloration—the trademark of this species—through digital photography, and compared male colours according to their age (juvenile versus subadult versus adult), group status (OMU holder versus bachelor), harem size (number of mature females) and the current season (mating versus non-mating). Sexual selection theory and previous studies of primate species in which males compete aggressively for social status lead us to specific testable predictions for the function of male coloration in black and white snub-nosed monkeys. These are that male coloration should be expressed more strongly in

(1) prime (adult) versus non-prime aged (juvenile, subadult) males,
(2) OMU holders versus bachelor males,
(3) males with larger harems, and
(4) the mating season versus the non-mating season;

We also tested for an interaction between the effect of age and season as well as group-holding status (harem holder versus bachelor) and season.

## 2. Material and methods

### 2.1 Data collection

We collected data on a semi-provisioned group of *Rhinopithecus bieti* in Xiangguqing, Golden Monkey National Park, Baimaxueshan Nature Reserve, Yunnan, China. The band consisted of 8 OMUs and peripheral bachelor males in AMUs. Individuals were fully habituated, fed daily with lichen, apples and other seasonally available items such as bamboo shoots, and were observed at distances ranging from 10 to 30 m.

The 15 males observed in this study were divided into bachelor (AMU) and OMU males. Eight males were associated exclusively with the AMU, four exclusively with OMUs, and three predominantly with OMUs but also with the AMU (i.e. either prior to gaining OMU residence status or after losing OMU residence status). No data were obtained for one OMU (‘Honglian’). All OMU males were fully adult (more than 8 years old), whereas bachelor males fell into different age categories: juvenile (*n*=2), subadult (*n*=2) and adult (*n*=2); two bachelor males transitioned from subadult to adult during the study. The number of adult females per OMU varied from 2 to 5. Photographs were taken between September 2012 and October 2013. The mating season lasted from August to October. We collected 27 photos taken in the mating season, 19 in 2012 and eight in 2013. All photos from the non-mating season were taken from November 2012 to July 2013. Photos from the non-mating season were available for all males in the sample, whereas for two males no photos were available from the mating season. The average number of photos per male from the mating season was 1.8 (range: 0–7, s.d. = 1.74), and from the non-mating season 4.9 (range: 1–8, s.d. = 2.17) (table 1).

**Table 1.** Total number of photographs in each season for each individual. AM, adult male; SAM, subadult male; JUV, juvenile male.

individual	age class	status	no. photos in mating season	no. photos in non-mating season	total no. photos
Bailian	AM	OMU/AMU^*b*^	4	8	12
Dagezi	AM	OMU	2	6	8
Dahuazui	AM	OMU/AMU^*b*^	0	2	2
Danba	AM	OMU/AMU^*b*^	1	3	4
Hongdian	SAM	AMU	2	3	5
Honglian	AM	AMU	0	4	4
Huachun	AM	OMU	7	6	13
Huangmao	SAM/AM^*a*^	AMU	1	8	9
Lianheguo	AM	OMU	2	5	7
Liebi	AM/SAM^*a*^	AMU	2	8	10
Liechun	JUV	AMU	1	1	2
Mili	JUV	AMU	1	4	5
Pianguan	AM	AMU	1	4	5
Rouliu	SAM	AMU	1	5	6
Yidianhong	AM	OMU	2	6	8

^*a*^The predominant age class is listed first.

^*b*^The predominant status is listed first.

### 2.2 Colour measurements

Photographs were taken with a Canon EOS 40D body and a Canon EF 70–200 mm 4/L IS USM lens. We selected images that were in focus, under diffuse lighting, and where the subject's face was in an approximately frontal orientation with respect to the camera from an average distance of 5 m. We only used images taken more than 5 min apart when the subject was in a different body position. Images were taken for another project and therefore did not use a protocol that we might normally use for assessing coloration. Images were taken in JPEG format in true-colour (24-bit) using the sRGB colour space, and so were subject to the camera's on-board JPEG compression and gamma encoding [26]. In addition to the absence of data on the spectral sensitivities of the camera sensors, and of snub-nosed monkey retinal receptors, this made accurate mapping of camera space to snub-nosed monkey colour space [27] impossible. A colour reference standard was also not included in images (or sequentially, [27]) preventing us from taking absolute colour measurements. We overcame these issues by instead measuring the relative colour contrast between the prominent red lip colour and the pale face colour, effectively standardizing the colour against face colour rather than a neutral photographic standard. While this does mean that lip colour is dependent on face colour: (i) we notice little inter-image or inter-individual variation in face colour and (ii) this may in any case be the more ecologically and behaviourally relevant measure, because for primates, colour contrasts provide a more reliable colour signal across environmental conditions than absolute colour does [28].

In total, we analysed samples from 100 images (mean: 6.67 per individual, s.d. = 3.15). We took colour measurements by segmenting out the lower lip and the face region immediately above the upper lip (figures 1 and 2). These two areas were chosen because they have similar orientations with respect to the light source, minimizing differences in colour measurements due to shading of the three-dimensional surface. We excluded all images where it was obvious the face and lower lip were not evenly lit (such as when the monkey was looking towards the ground). When cropping we also avoided selecting areas that were in shadow. After selecting the pixels in the two regions, we decompressed RGB values using functions in MATLAB so that they were approximately linear with respect to light intensity (using sRGB standard encoding and decoding gamma). We then calculated the mean RGB values of each patch and divided the lip colour by the face colour to correct for differences between images in the colour of the ambient light and the white point used by the camera when taking the photograph (by effectively setting the face colour as the white point). We then reapplied the gamma correction and converted from sRGB colour space to L*a*b* colour space. L*a*b* colour space is designed to be perceptually uniform (for the human visual system) so each unit change corresponds to an equal change in perceived visual difference. The L* component corresponds to lightness and the a* and b* components to two colour opponent channels with a* spanning the redness to greenness of a stimulus and b* spanning the blueness to yellowness of the lip colour.

We tested the reliability of colour measurements by identifying pairs of photos for each individual taken on the same day, but at least 5 min apart. Nine pairs of photographs met these criteria. Intraclass correlation coefficients were high and significantly different from zero for L* (R2=0.927, *F*_7,8_=26.25, *p*<0.001, 95% CI [0.71,0.98]), a* (R2=0.957, *F*_7,8_=46.59, *p*<0.001, 95% CI [0.82,0.99]) and b* (R2=0.776, *F*_7,8_=7.91, *p*=0.005, 95% CI [0.27,0.95]), indicating that measurements of the same individuals were highly consistent across repeat samples.

### 2.3 Data analysis

We computed an LMM with age (juvenile, subadult and adult), male reproductive status (OMU versus AMU), number of harem females, mating season (yes versus no), and the interaction between age and season as fixed effects and redness (a*) as the response variable. A second LMM included the same variables, but the interaction age : season was replaced by status : season. To test that status effects were not influenced by the inclusion of juvenile individuals, we also undertook the second model with juvenile individuals excluded. The second model was also run with lightness (L*) as the response variable. ‘Individual’ was included as a random effect in all models. Parameter-specific *p*-values were approximated using normal distribution. All analyses were conducted using the lme4 package in R statistical software v. 3.1.0 (The R Foundation for Statistical Computing, Vienna, Austria, http://www.r-project.org).

## 3. Results

**Figure 3.**
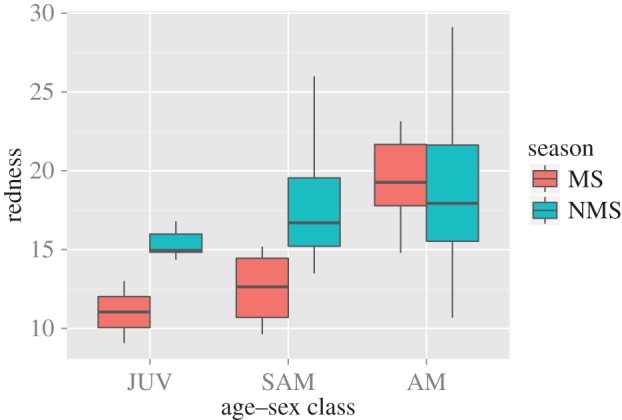
Levels of lip redness in different age–sex classes of *Rhinopithecus bieti* males in the mating (MS) and non-mating season (NMS).

**Figure 4.**
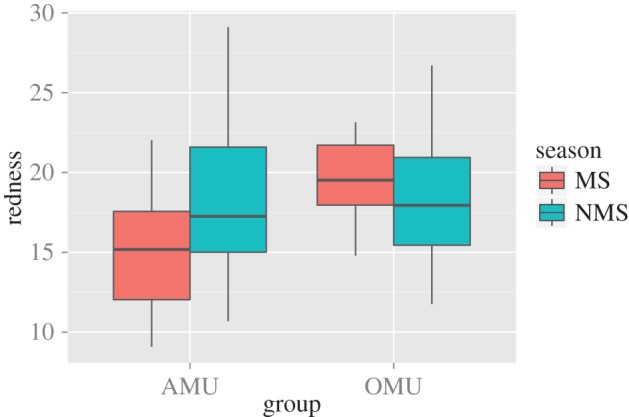
Levels of lip redness for AMU and OMU males outside (NMS) and during the mating season (MS).

We found increasing lip redness with age (tables 2 and 3; figure 3). The interaction season : age had a significant effect on redness, indicating that juvenile and subadult males become less red in the mating season (table 2 and figure 3). The interaction between group-holding status and mating season was significant, regardless of whether all age categories were included in the model (table 3) or juveniles were excluded (electronic supplementary material, table S1). Plotting shows that during the non-mating season, AMU and OMU males do not differ in lip redness. However, during the mating season, colour intensity for OMU males is much higher than for AMU males (figure 4). Plotting also shows that the result for the interaction effect in this model is essentially being driven by a fading of colour in the non-adult AMU males, in combination with a reddening of the OMU holders (figure 4). Lip lightness was not influenced by any of the predictor variables tested (electronic supplementary material, table S2).

**Table 2.** Model of the effects of reproductive status, age, number of group females, season and the interaction season : age on redness of male lip colour. Significant *p*-values are highlighted in bold. Variables included in significant interaction terms cannot be interpreted as independent variables (in italics).

	estimate	s.e.	*t*-value	*p*-value
intercept	19.126	1.21	15.75	0
mating season (no)	−0.638	0.91	−0.70	*0.484*
age (juvenile)	−8.084	3.02	−2.68	*0.007*
age (subadult)	−6.699	2.16	−3.10	*0.002*
status (OMU)	−0.986	2.40	−0.41	0.681
no. females	0.401	0.85	0.47	0.638
mating season (no) : age (juvenile)	5.140	3.02	1.70	0.089
mating season (no) : age (subadult)	6.297	2.17	2.90	**0.004**

**Table 3.** Model of the effects of reproductive status, age, number of group females, season and the interaction season : status on redness of male lip colour. Significant *p*-values are highlighted in bold. Variables included in significant interaction terms cannot be interpreted as independent variables (in italics).

	estimate	s.e.	*t*-value	*p*-value
intercept	16.603	1.36	12.19	0
mating season (no)	3.768	1.19	3.18	*0.002*
age (juvenile)	−5.507	2.16	−2.55	**0.011**
age (subadult)	−3.577	1.29	−2.77	**0.006**
status (OMU)	2.007	2.69	0.75	*0.456*
no. females	0.394	0.84	0.47	0.641
mating season (no) : status (OMU)	−5.768	1.61	−3.58	<**0.001**

## 4. Discussion

In line with studies of geladas [8], our results support the prediction that group-holding males display greater reddening of sex skin. Males in OMUs who possess near universal monopolization of sexually mature females scored higher on lip redness than bachelor males. Lip redness may thus be an indicator (‘badge’) of male social status (*sensu* [29]). However, this effect was dependent on season; coloration intensity was stronger in the mating season when harem holders benefit from displaying to (unfamiliar) bachelor males their ability and willingness to defend their unit [30]. It is also possible that these signals are influenced by selection through mate choice decisions of resident or non-resident females, though behavioural data on female mate choice would be needed to test this.

Group-holding status interacted with season to affect coloration, with OMU leaders turning redder in the mating season and bachelor males fading in redness during that period. In mandrills, males do not show marked seasonal changes in sexual skin coloration [31], but changes in male coloration temporally tied to increases in female fertility have been reported in both seasonally breeding primates such as rhesus macaques [22], and aseasonal breeders such as crested macaques [23]. One interpretation of our findings is that by advertising coloration OMU males may be indicating their social status to rivals, and potentially also their ability and willingness to defend their OMU from other males. The weakening of red colour intensity exhibited by non-adult bachelor males can perhaps be seen as ‘social camouflage’ in which they mask their maleness during the period of reproductive competition to avoid conflict. Data for geladas [8] show that OMU holding males are more colourful than bachelor males, even on the day of successful takeover challenges. As such, coloration clearly does not indicate competitive ability *per se* in geladas—challenger males that were able to outcompete resident males were less colourful on the day of the challenge [8]. If the challenge is successful, coloration changes then follow, with deposed males losing colour, and new OMU holders gaining colour. This is also consistent with data on mandrills, which show that coloration changes in response to (rather than in advance of) dominance changes [6,7]. As such, coloration in these species seems to indicate social status, specifically.

Using OMU size as a measure of male success in attracting females, we found no evidence that females prefer more brightly coloured males [13]. For geladas, Bergman *et al.* [8] argued that ‘it seems unlikely that a leader male would need to advertise his quality to the individuals that are most familiar with him—mainly the females in his unit’ (p. 804). In a society where female choice is manifest, as in the bisexual-dispersal system of *Rhinopithecus*, females may use male colour cues when selecting a breeding unit, but other factors are also certainly involved [32,33]. An intriguing way of further exploring the female attraction hypothesis would be to examine the choices females make when selecting a male for extra-unit copulations, which have been observed on several occasions in the study population (P. Zhu 2013, unpublished data) and also reported for the closely related *Rhinopithecus roxellana* [34]. Another possibility related to female mate choice is that males might be using colour signals to try to entice females from coresident OMUs to transfer to their unit or cajole them into an extra-unit copulation. The former seems less likely as adult females (at least in*R. roxellana*) do not typically transfer in the mating season, but in the period between the birth and mating season [33]. The latter is a possibility considering that extra-unit copulations seem to be concentrated in the mating season (in the better studied *R. roxellana*) [34,35].

It is unknown if male dominance between different OMUs impacts skin coloration. Data on approach–retreat interactions, required for establishing dominance relations among OMU leaders, are currently unavailable. In this study, there was no effect of unit size (i.e. the number of females) on lip redness, but it is unclear if unit size is a good proxy for dominance; tenure length (as a measure of resilience to takeover attempts) might be a better measure here, but such data are unavailable. Variation in number of females per OMU was also small (ranging from 2 to 5), which makes the detection of statistically significant differences difficult. In *R. roxellana*, featuring a similar social organization, OMUs are ranked hierarchically [36]. Both the size of OMUs and the duration of their presence in the band have been shown to influence male dominance rank [36], but it is uncertain which variable has better predictive power. If differences between males of different units were related to differences in their fighting abilities, it could be that males use red coloration as a condition-dependent and reliable trait to signal to rivals [6], which guides them in deciding which unit holder to challenge, and prevents ‘unnecessary’ conflict between leaders and bachelors [8,37].

Female *R. bieti* also have conspicuous red lips which could reflect underlying intrasexual competition over access to males in social units containing several females, but this would require further study. Competition among females over males (*sensu* [38]) in *Rhinopithecus* is expressed in the form of sexual interference during mating by co-residing females [39]. Red lip coloration in females may constitute a sexually selected ornament which signals variation in fecundity/reproductive quality and is thus subject to male mate choice [40]. They may also undergo temporal changes in line with reproductive receptivity akin to the expression of sexual swellings in some primates [41]. In rhesus [42–44] and Japanese macaques [45], females undergo facial colour changes that coincide with the fertile phase of the cycle.

In conclusion, our data indicate that male coloration appears to be a sexually selected trait as it is related to reproductive competition. However, our data are agnostic on whether the mechanisms of selection are likely to revolve around male–male competition, female mate choice or both. Behavioural data are needed to distinguish between these potential mechanisms. Future work should also aim to collect a larger sample of images with a calibrated camera set-up that would allow data to be mapped from the camera's colour space directly to a primate perceptual colour space [43]. Other further work might address the proximate hormonal mechanisms, such as androgen concentrations, which might underlie the expression of the trait [46].

## Supplementary Material

File 1: Supplementary material - Data

## Supplementary Material

File 2: Supplementary material – Tables

## Data accessibility

The datasets supporting this article have been uploaded as part of the electronic supplementary material. The first worksheet contains the raw data and the second worksheet contains all the variables used in the statistical model.
