# Supplementary material for: Sexually selected lip colour indicates male group-holding status in the mating season in a multi-level primate society
Source: R Soc Open Sci. 2015 Dec 16;2(12):150490. doi: 10.1098/rsos.150490 (PMC4807456; doi:10.1098/rsos.150490)
Supplement: File 2: Supplementary material – Tables [file rsos150490supp2.docx]

**Supplementary material – Tables**

**Table S1**. Model of the effects of reproductive status, age, number of group females and season and the interaction season:status on redness of male lip color. Juvenile males are excluded from this model.

|  | **Estimate** | **Standard error** | **t value** | **p** |
| --- | --- | --- | --- | --- |
| Intercept | 16.042 | 1.48 | 10.86 | 0 |
| **Mating season (no)** | 3.115 | 1.32 | 2.36 | *0.018* |
| **Age (subadult)** | -1.632 | 1.35 | -1.21 | 0.226 |
| **Status (OMU)** | 2.554 | 2.91 | 0.88 | *0.380* |
| **# females** | 0.382 | 0.92 | 0.42 | 0.678 |
| **Mating season (no):status (OMU)** | -4.470 | 1.73 | -2.59 | **0.01** |

Significant p values are highlighted in bold. Variables included in significant interaction terms cannot be interpreted as independent variables (in italics).

**Table S2**. Model of the effects of reproductive status, age, number of group females and season and the interaction season:status on lightness of male lip color.

|  | **Estimate** | **Standard error** | **t value** | **p** |
| --- | --- | --- | --- | --- |
| Intercept | 67.466 | 2.87 | 23.53 | 0 |
| **Mating season (no)** | -0.696 | 2.43 | -0.29 | *0.775* |
| **Age (juvenile)** | 7.687 | 4.73 | 1.63 | *0.104* |
| **Age (subadult)** | -2.836 | 2.73 | -1.04 | 0.299 |
| **Status (OMU)** | 2.221 | 5.74 | 0.39 | *0.699* |
| **# females** | -0.905 | 1.84 | -0.49 | 0.623 |
| **Mating season (no):status (OMU)** | -0.913 | 3.31 | -0.28 | 0.782 |

Significant p values are highlighted in bold. Variables included in significant interaction terms cannot be interpreted as independent variables (in italics).
